# The Effect of Dictation on Emergency Medicine Resident Time to Note Completion

**DOI:** 10.5811/westjem.41812

**Published:** 2025-11-17

**Authors:** Lauren R. Willoughby, Daniel J. Hekman, Benjamin H. Schnapp

**Affiliations:** * Medical College of Wisconsin, Department of Emergency Medicine, Milwaukee, Wisconsin; † University of Wisconsin, Department of Emergency Medicine, Madison, Wisconsin

## Abstract

**Introduction:**

Timely documentation of a patient encounter is a necessary component for delivering high-quality healthcare as it has direct impacts on continuity of care. The use of voice recognition software has been integrated into the electronic health record (EHR) to increase efficiency of documentation. We aimed to investigate the impact of dictation use on emergency medicine (EM) residents’ time to note completion.

**Methods:**

We conducted this study in a three-year EM residency program at an academic emergency department. Notes written in the EHR by EM residents were included for analysis. We split notes into two cohorts based on academic year: 2018–19 academic year (AY18–19); and 2021–22 academic year (AY21–22). We analyzed approximately 37,000 notes per cohort. Dictation was available to all residents in each cohort. The length of the note (measured by character count) and time to note completion (less than or greater than 24 hours) was analyzed.

**Results:**

For both the AY18–19 and AY21–22, the rate of note completion within 24 hours was higher when using dictation compared to typing (odds ratio [OR] 1.3 and OR 2.9, respectively). Aggregated data of both cohorts showed 77.9% of dictated notes were completed within 24 hours compared to 70.9% of typed notes (*P* < .001). In both cohorts, the average number of characters per note was larger if the note was dictated. For AY18–19, the average was 6,628 characters for dictated notes vs 6,136 for typed notes (*P* < .05). Similarly, for AY21–22, the average was 6,531 vs 6,347 (*P* < .05).

**Conclusion:**

The use of dictation by EM residents for note completion resulted in a higher likelihood of the note being completed within 24 hours.

## INTRODUCTION

Timely documentation of a patient encounter is a necessary component for delivering high-quality healthcare as it has a direct impact on continuity of care.^1^,^2^ However, many physicians report being unable to complete tasks related to the electronic health record (EHR) during their clinical hours, often requiring remote access to the EHR outside scheduled work time.^3^ Inefficient documentation processes may contribute to physicians’ spending significant time outside their clinical shifts to finalize patient notes.

Initiatives aimed at improving physician well-being have targeted the reduction of EHR- and documentation-related burdens.^4^ The Stanford Model of Occupational Well-Being is a widely recognized framework that identifies workplace efficiency as a component influencing physician wellness.^5^ Within this model, streamlined documentation is highlighted as a critical factor in enhancing workplace efficiency. Therefore, optimizing workplace efficiency by improving interactions with the EHR may support physician wellness. Additionally, the Council of Residency Directors in Emergency Medicine has recommended optimization of the EHR as a best practice for promoting resident wellness.^6^

Various interventions have been tried to decrease the burden of charting on clinicians. To help increase the efficiency of documentation and decrease the amount of time spent on documenting, some EHR vendors have introduced shortcuts, often called “dot phrases,” which are short texts beginning with “.” that are typed into a note and are then replaced by different, often more lengthy, text (for example, replacing “.wdl” with “within defined limits”). However, a recent study has shown that dot phrases do not affect the time to note completion.^7^ The use of a medical scribe, a person who accompanies the physician during the patient encounter and documents portions of the chart, has been shown to increase clinician efficiency.^8^ One study demonstrated that faculty use of scribes can enhance resident educational experiences.^9^ However, there is a lack of research examining the effects of scribes documenting on behalf of residents, specifically regarding the impact on a resident’s ability to independently and effectively document in the EHR. Another strategy to alleviate the burden of documentation is the integration of voice recognition software. The use of dictation strongly correlated with increased emergency medicine (EM) resident productivity as measured by relative value units per hour.^10^

However, to our knowledge, there has not been a study investigating the effect of dictation on timely EM resident note completion as an indicator of efficiency. Our primary goal in this study was to determine whether dictated notes were linked to improved documentation efficiency by assessing whether notes are more likely to be completed within 24 hours when dictation is used.

## METHODS

### Study Setting and Population

The study was conducted in a three-year EM residency program at an academic emergency department (ED). The ED is in a midwestern, small-city urban location with 54 beds and approximately 60,000 patient visits annually. The EM residents were eligible for inclusion if they had worked in the ED uninterrupted between 2017–2022. There were 12 EM residents per year until 2020, after which there were 13 per year. The institution uses M*Modal Fluency Direct (3M Company, Maplewood, MN) for dictation, and a microphone and dictation software integrated with the EHR was available at each workstation and in multiple shared work areas (eg, resident lounge). All residents receive dictation training during residency orientation.

Most residents will start a patient note while on shift, but it is not mandatory that they stay at the hospital after their shift to complete their notes. All residents receive personal microphones, can dictate using their personal phone, and can access dictation software remotely; thus, they are able to complete notes at their convenience. Additionally, there is no strict consequence for not completing a note within 24 hours. However, weekly reports are generated, and residents who are frequently delinquent may be placed on an individualized improvement plan or probation based on tardy chart completion.

**Population Health Research Capsule**

What do we already know about this issue?

*Timely documentation is crucial to high-quality patient care as it facilitates continuity among team members.*

What was the research question?

*We examined whether the use of dictation improves the timeliness of documentation completion.*

What was the major inding of the study?

*Within two cohorts analyzed, dictation improved timely note completion, with ORs of 1.47 and 2.93, respectively.*

How does this improve population health?

*Using dictation results in faster time to note completion, enhancing continuity of patient care and supporting clinician wellness by reducing the amount of after-hours work.*

### Study Protocol

We included notes written by EM residents between 2017–2022. This included notes from an adult ED at an academic medical facility, a pediatric ED, and an affiliated community ED. Although a randomized control study design was considered, we conducted a retrospective study to better reflect real-world use of dictation in actual clinical practice. Data obtained included the note length (total number of characters in the note), whether the note was signed by a resident within 24 hours of the resident assigning him/herself to the patient, and whether dictation was used to complete any portion of the note. We collected data on note length to assess whether it acted as a confounder, specifically examining whether shorter notes are associated with faster chart completion. For this study, we defined efficiency as documentation timeliness, measured by the completion of notes within 24 hours. This timeframe was selected as it aligns with institutional documentation standards and logically supports continuity of care among the healthcare team. We did not assess quality of the note, although this is also important.

Each resident was assigned a study identification number, and the identification key was accessible only by the data scientist on the study team (DH) who did not perform the statistical analysis. Patient information was de-identified for the author performing the data analysis (LW). Data was imported to Microsoft Excel (Microsoft Corp, Redmond, WA) for analysis.

### Data Analysis

Analysis was run on notes completed by two cohorts of residents: the 2018–19 academic year (AY18–19) and the 2021–22 academic year (AY21–22). We excluded AY19–20 and AY20–21 due to the effects of the COVID-19 pandemic on clinical volumes and related interruptions to clinical practice. Notes that included any portion completed using dictation were identified by an internal flag within the EHR. We stratified notes by disposition to account for the fact that some notes may be completed sooner if they have a higher priority disposition. For example, the note for a patient being transferred to an outside facility needs to be completed before transfer. Each patient note was coded into one of two categories based on their disposition: high priority (“Admit,” “Transfer to another facility,” “Against medical advice”); or regular priority (all other disposition codes that were not a high priority). Notes for patients handed off during shift change, or “sign out” patients, were not categorized as high priority.

We used a logistic regression model to adjust for covariates potentially affecting time to note completion. Predictor variables included dictation use, note length, resident’s postgraduate year (PGY), and note priority. Additionally, one resident was excluded from the analysis due to an off-schedule progression through the program and graduation. This study was deemed exempt as quality improvement by the institutional review board.

## RESULTS

We included 37,778 notes in the analysis of the AY18–19 cohort and 37,716 notes of the AY21–22 cohort. Of the 37,778 notes in the AY18–19 cohort, 35,162 (93.08%) were non-priority dispositions and 2,616 (6.92%) were high-priority dispositions. Of 37,716 notes in the AY21–22 cohort, 36,692 (97.28%) were non-priority and 1,024 (2.72%) were high priority.

In the AY18–19 cohort, the unadjusted rate of note completion within 24 hours was higher when using dictation compared to typing (odds ratio [OR] 1.3). Similarly, in the AY21–22 the unadjusted rate of note completion within 24 hours was higher when using dictation compared to typing (OR 2.9). Aggerated data from the two cohorts showed that 77.9% of dictated notes were completed within 24 hours vs 70.9% of typed notes (*P* < .001). The Figure depicts the overall aggregated time to note completion for both academic years with and without dictation. In both the AY18–19 and AY21–22 cohorts, the logistic regression model was statistically significant, and all the predictors were significant as well. Additionally, the AY18–19 model correctly predicted timely note completion approximately 60% of the time, and the AY21–22 model correctly predicted completion of notes in 24 hours in the analysis sample approximately 68% of the time. The *R*^2^ for AY18–19 was .0453, and the *R*^2^ for AY21–22 was .1151.

In the AY18–19 cohort, dictation use and disposition priority status were both strong positive predictors of timely note completion while an increase in note length, whether dictated or typed, was associated with a decrease in the likelihood of timely note completion. The predicted probability of an average length, regular-priority note being completed on time by a PGY-3 resident when dictation was not used was 0.63, and 0.71 when dictation was used (OR 1.47). Timely note completion was about one and a half times more likely with dictation than without after controlling for other explanatory variables.

In AY21–22, most of the same effects were observed, with dictation use predicting an even greater increase in the likelihood of timely note completion. After adjusting for other predictors, the OR for note completion within 24 hours with dictation use was 2.93, aligning with the simple contingency analysis. In the AY18–19, the average number of characters per typed note was 6,136 compared to dictated notes, which was significantly larger at 6,628 (*P* < .05). In the AY21–22, the average number of characters per typed note was 6,347; the average number of characters per dictated note was significantly larger at 6,531 (*P* < .05).

## DISCUSSION

Dictation is widely used among EM residents—at least in this residency program. In both cohorts analyzed, the rate of note completion within 24 hours was more likely with dictation than without after controlling for other explanatory variables. The use of dictation improving documentation efficiency is a crucial finding as other interventions, such as dot phrases, have not been proven to increase efficiency.^6^ (Interestingly, the integration of artificial intelligence may enhance efficiency of documentation; however, this is in still in the preliminary stages of evaluation.^11^) The relatively low *R*^2^ suggests that even though dictation and the other predictors have statistically significant relationships with timely note completion, many external factors played an important role in note completion, which our study was not able to capture. Additionally, there is a noteworthy difference in the ORs between the two cohorts with respect to time to note completion and note length. The reason for these differences is unclear and warrants further investigation, potentially through focus groups or qualitative methods, to explore differences in workflows, training, or other contextual factors between the resident cohorts.

While dictation use was associated with a higher rate of note completion within 24 hours, it did result in longer note length by several hundred characters. It is important to acknowledge that while the note length was longer in dictated notes, we did not assess the quality of included information. It is possible that the additional included information was beneficial and that dictation facilitates easier inclusion of helpful medical information and decision-making. However, it is also possible that dictation results in a greater volume of low-quality information being included in the note. Significant inter-physician variation has been previously noted in EHR data and has been cited as a possible barrier to safe and efficient care.^12^ Assessment of the quality of dictated vs typed notes represents an avenue for further study. Additionally, dictation is also known to cause errors in transcription, some of which can be clinically significant.^13^ Exploring the impact of these errors on resident documentation efficiency and clinical care could also be investigated further.

Finally, the association between dictation and increased note completion within 24 hours suggests potential downstream effects on resident wellness, as it may contribute to greater workplace efficiency and reduce the need to complete notes outside clinical shifts. This relationship warrants further investigation.

## LIMITATIONS

There are several limitations to this study. First, because we conducted this study at a single institution generalizability may be limited; other implementations of dictation could provide different results. Additionally, notes were included for analysis if any portion of the note was completed with dictation, but there was no data on how much of the note was completed with dictation. Further, not all notes written by residents were included for analysis. Notes written at the Veterans’ Administration hospital and the unaffiliated community site were not included as these sites represented a small minority of the residents’ training experience and used a different EHR. Finally, factors that may influence time to note completion, such as priority of disposition, were accounted for; however, there are additional factors that may impact time to note completion that were not accounted for, such as patient volume or overall shift workload. Future areas of investigation may include conducting focus groups or interviews to further investigate the perception of residents on what factors influence time to note completion and how dictation influences efficiency.

## CONCLUSION

The use of dictation by residents for note completion results in a higher likelihood of the note being completed within 24 hours.

## Figures and Tables

**Figure 1:**
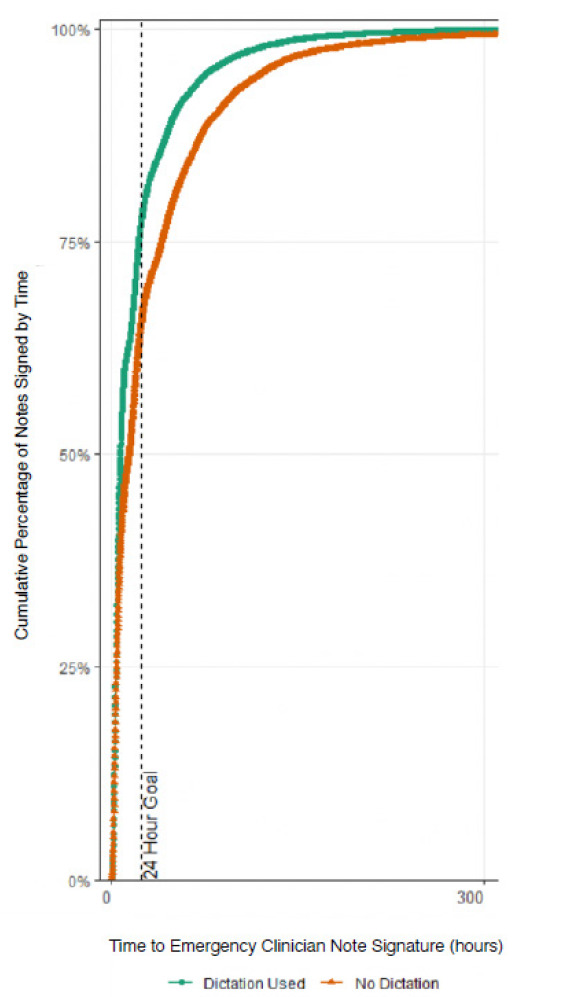
Overall aggregated time to note completion by emergency medicine residents for academic years 2018–2019 and 2021–2022 with and without dictation.
